# Lung transplantation from polytrauma donors: beyond primary graft dysfunction

**DOI:** 10.3389/fmed.2026.1851797

**Published:** 2026-06-19

**Authors:** Chiara Catelli, Daniele Marianello, Andrea Lloret Madrid, Marianna Rizzo, Miriana D'Alessandro, Margherita Sambo, Francesca Montagnani, Marco Guerrieri, David Bennett, Elena Bargagli, Piero Paladini, Federico Franchi, Luca Luzzi

**Affiliations:** 1 Thoracic Surgery and Lung Transplant Unit, Department of Medicine, Surgery and Neurosciences, University of Siena, Siena, Italy; 2 Cardiothoracic and Vascular Anesthesia and Intensive Care Unit, Department of Medicine, Surgery and Neurosciences, University of Siena, Siena, Italy; 3 Department of Life Sciences, Health and Healthcare Professions, Link Campus University, Rome, Italy; 4 Infectious and Tropical Diseases Unit, Department of Medical Biotechnologies, University of Siena, Siena, Italy; 5 Respiratory Diseases Unit, Department of Medicine, Surgery and Neurosciences, University of Siena, Siena, Italy

**Keywords:** donor pool, lung transplantation, polytrauma, primary graft dysfunction, pulmonary function

## Abstract

**Background:**

Lung donors are increasingly older and marginal. Polytrauma donors (PD) represent a potentially valuable resource due to their younger age, although trauma-related lung injury raises concerns regarding graft quality. This study aimed to evaluate early and mid-term outcomes after lung transplantation using lungs from PDs compared with non-polytrauma donors (NPD).

**Methods:**

We retrospectively analyzed 125 lung transplant recipients from brain-dead donors between January 2013 and June 2024. Perioperative outcomes, primary graft dysfunction (PGD), and pulmonary function at 1, 3, and 6 months were compared between PD (*n* = 36) and NPD (*n* = 89) groups. To account for baseline differences, a 1:1 propensity score matching (PSM) was performed based on donor age, recipient age, and ischemic time.

**Results:**

In the overall cohort, PDs were younger and more frequently male, with a higher prevalence of pulmonary contusions. Early postoperative outcomes, including PGD, ICU stay, and perioperative complications, were comparable between groups. At 6 months, overall survival was higher in the PD group (91.7 vs. 75.3%, *p* = 0.048). After PSM, no significant differences were observed in survival (*p* = 0.66), perioperative outcomes, or pulmonary function. Functional recovery and CLAD rates were similar between groups across all time points.

**Conclusions:**

Lung transplantation using grafts from polytrauma donors is not associated with worse perioperative or mid-term outcomes. After adjustment for baseline differences, outcomes were comparable to those from non-polytrauma donors, supporting the safe and selective use of these grafts as a strategy to expand the donor pool.

## 1 Introduction

Lung transplantation (LTX) remains limited by the scarcity of suitable organs, driving interest in extended-criteria donors (1). Among these, donors who die following polytrauma represent a substantial but underutilized resource. Several studies demonstrate that polytrauma donors (PD) are typically younger, with fewer comorbidities and shorter hospital stays, characteristics that would theoretically favor organ quality (2). Despite this favorable profile, lungs from PD are disproportionately discarded, most often because of radiologic pulmonary contusions, common sequelae of blunt thoracic injury. Traditional donor-selection guidelines consider pulmonary contusions as relative contraindications for lung procurement due to concerns about primary graft dysfunction (PGD) (3, 4). Pulmonary contusion reflects direct parenchymal injury that can compromise lung mechanics and oxygenation, while the cumulative insult in PD, including chest trauma, transfusion-related lung injury, aspiration, and barotrauma, creates a pro-inflammatory state that may prime the lung for further injury (5). Elevated inflammatory mediators and endothelial activation have been associated with increased risk of PGD (6). Although emerging evidence suggests that lungs from PD, even when radiologic contusions are present (7), can yield outcomes comparable to those of non-trauma donors (NPD), most studies focus solely on PGD. Broader perioperative parameters and postoperative pulmonary function are rarely evaluated despite their clinical relevance, although they are frequently associated with the development of PGD but may also occur independently of it (8). The objective of this study is to evaluate early and mid-term outcomes after lung transplantation using organs from PD, with particular focus on perioperative course and postoperative pulmonary function, compared with NPD.

## 2 Materials and methods

This retrospective observational study was designed and reported according to the STROBE guidelines. The study was conducted in accordance with the Declaration of Helsinki and the principles of the ISHLT Statement on Transplant Ethics, and was approved by the Ethics Committee of the University of Siena. This study included all consecutive patients who underwent LTX at the Thoracic Surgery and Lung Transplant Unit of the Siena University Hospital between January 2013 and June 2024. Only recipients with complete donor-related data were included in the study. To minimize bias related to donor type, recipients who received lungs from donors after circulatory death (DCD) were excluded. In addition, recipients who died during the surgical procedure were excluded to allow meaningful analysis of perioperative outcomes. Written informed consent for participation in the study was obtained from all transplant recipients. Autonomous consent free from coercion was obtained from the donors or their next of kin; organs were not sourced from executed prisoners or prisoners of conscience.

### 2.1 Donor evaluation

Donor data were retrieved from the Tuscan regional online database, which includes all donors proposed to our center, both intra- and extra-regionally. Collected variables included donor age, cause of death, smoking history, duration of mechanical ventilation, bronchoscopic findings, arterial oxygenation expressed as the PaO_2_/FiO_2_ ratio at the time of evaluation, history of cardiopulmonary resuscitation, and evidence of aspiration. Donors were classified as polytrauma if death resulted from multiple traumatic injuries involving at least two body regions including the chest, with at least one being potentially life-threatening. In PDs, available chest radiographs and/or computed tomography reports were retrospectively reviewed to assess the presence of pulmonary parenchymal consolidations. For each donor, the Oto lung donor score (9) was calculated. The use of *ex vivo* lung perfusion (EVLP) was reserved for selected donor lungs considered at higher risk of dysfunction based on clinical and functional criteria, in accordance with institutional protocols (10). However, the presence of significant pulmonary contusions was considered a contraindication to EVLP in our center, due to concerns regarding exacerbation of trauma-related lung injury during reperfusion. As a result, lungs from polytrauma donors were not subjected to EVLP.

### 2.2 Recipient characteristics

Graft allocation at our center follows the Lung Allocation Score (LAS) system (11). Based on donor trauma status, recipients were assigned to either the PD group or the NPD group. Recipient data included preoperative clinical characteristics, intraoperative and postoperative outcomes. Perioperative data comprised duration of mechanical ventilation, length of ICU-stay, need for extracorporeal membrane oxygenation (ECMO), postoperative lactate trends, development of acute kidney injury (AKI), need for tracheostomy, use of continuous renal replacement therapy (CRRT). Inotropic score (12) was used as a composite indicator of postoperative hemodynamic support and was calculated according to standard formulas as follows: dopamine dose (μg/kg/min) + dobutamine dose (μg/kg/min) + 100 × epinephrine dose (μg/kg/min) + 100 × norepinephrine dose (μg/kg/min) + 10 × milrinone dose (μg/kg/min). PGD was assessed and graded at 72 h after transplantation according to the International Society for Heart and Lung Transplantation (ISHLT) criteria (13). Postoperative surgical airway complications were also recorded based on clinical and radiologic evidence. Post-transplant pulmonary function was evaluated whenever feasible using spirometry at 1, 3, and 6 months after transplantation, including forced expiratory volume in one second (FEV_1_), forced vital capacity (FVC), vital capacity (VC), total lung capacity (TLC) and diffusing capacity for carbon monoxide (DLCO). Overall survival (OS) was calculated from the date of transplantation to death from any cause or last follow-up. Follow-up analyses were focused on the first 6 months after transplantation, as this time frame captures clinically meaningful early and mid-term outcomes while minimizing the impact of confounding factors related to possible long-term complications. The surgical technique and postoperative medical management were standardized throughout the study period and have been described in detail elsewhere (14, 15).

### 2.3 Statistical analysis

Descriptive statistics were used to summarize the baseline characteristics of the study population. Categorical variables were expressed as absolute frequencies and percentages and compared using the Pearson's chi-squared test. Continuous variables were presented as medians with interquartile ranges (IQR). Comparisons between the two groups (PD vs. NPD) were performed using the non-parametric Mann-Whitney U test.

To address potential selection bias arising from baseline differences between the groups (particularly regarding donor and recipient age), a 1:1 Propensity Score Matching (PSM) was performed. The propensity score (PS) for each patient was calculated using a multivariable logistic regression model with donor_type as the dependent variable and donor age, recipient age, and ischemic time as covariates. Matching was executed using the nearest neighbor method without replacement, utilizing a caliper of 0.05 to ensure high-quality pairings. After matching, the balance of covariates was verified using independent *t*-tests, with a *p*-value > 0.05 indicating successful harmonization.

Graft survival analysis was performed using the Kaplan-Meier method, and differences between the survival curves of the two groups were evaluated using the Log-rank (Mantel-Cox) test to account for potential differences in early vs. late mortality.

To evaluate the independent impact of polytrauma on graft survival, a multivariable Cox proportional hazards regression was performed. The model was adjusted for key clinical confounders, including donor age, recipient age, and ischemic time. Effect sizes are reported as Hazard Ratios (HR) with 95% Confidence Intervals (CI). Missing data were handled through listwise deletion, as they occurred at random and affected a minimal percentage of cases. Statistical significance was defined as a *p*-value < 0.05. All statistical analyses were performed using Jamovi and GraphPad Prism software (Dotmatics, Boston, MA).

## 3 Results

### 3.1 Donor analysis

Between January 2013 and June 2024, 146 patients underwent LTX at our institution. Of these, 21 patients were excluded from the analysis: 3 DCDs, 2 intraoperative deaths, and 16 cases with missing donor data (specifically, donor-related variables were not available in the regional database because authorization for data sharing on the platform had not been granted by the donor's next of kin), resulting in a final study population of 125 recipients, of whom 36 (28.8%) were classified as PD and 89 (71.2%) as NPD. No patients were lost to follow-up during the study period. Donors' characteristics are shown in Table 1. PDs were significantly younger, with a median age of 31.5 years (IQR 18.8–48.2) compared with 52.0 years (IQR 44.0–61.0; *p* < 0.001). Female sex was less frequent among PD (25.0 vs. 57.3%; *p* = 0.002).

**Table 1:** Comparison of donor characteristics between polytrauma and non-polytrauma donors.

	Overall cases (*n* = 125)			Propensity score-matched pairs (*n* = 72)		
Variables	No Polytrauma	Polytrauma	*p*	No Polytrauma	Polytrauma	*p*
	*N* = 89	*N* = 36		*N* = 36	*N* = 36	
Age, years	52.0 (44.0–61.0)	31.5 (18.8–48.2)	< 0.001	43.5 (30.5–47.0)	31.5 (18.8–48.2)	0.193
Female sex, *n* (%)	51 (57.3)	9 (25.0)	0.002	22 (61.1)	9 (25.0)	0.004
Blood type, *n* (%)			0.042			0.258
A	40 (44.9)	15 (41.7)		17 (47.2)	15 (41.7)	
B	4 (4.5)	7 (19.4)		2 (5.6)	7 (19.4)	
0	42 (47.2)	14 (38.9)		16 (44.4)	14 (38.9)	
AB	3 (3.4)	0 (0.0)		1 (2.8)	0 (0.0)	
Height, cm	170.0 (165.0–175.0)	175.0 (170.0–180.0)	< 0.001	170.0 (165.0–177.8)	175.0 (170.0–180.0)	0.007
Weight, kg	70.0 (60.0–80.0)	75.0 (70.0–85.0)	0.011	67.5 (60.0–80.0)	75.0 (70.0–85.0)	0.065
Body Mass Index, kg/m^2^	23.5 (21.8–26.1)	24.2 (22.8–26.0)	0.447	23.3 (21.5–27.6)	24.4 (22.8–26.2)	0.398
Smoking history, *n* (%)			0.958			0.689
Never	67 (75.3)	27 (75.0)		25 (69.4)	27 (75.0)	
Current	16 (18.0)	7 (19.4)		7 (19.4)	7 (19.4)	
Former	6 (6.7)	2 (5.6)		4 (11.1)	2 (5.6)	
PaO_2_/FiO_2_ ratio	487.0 (441.0–535.0)	476.2 (433.7–550.5)	0.978	482.5 (418.8–543)	476.5 (434–550.5)	0.787
Pulmonary contusions, *n* (%)	1 (1.1)	34 (94.4)	< 0.001	0 (0.0)	34 (94.4)	< 0.001
Bronchoscopic secretions, n (%)	11 (12.5)	4 (11.4)	>0.99	5 (14.3)	4 (11.4)	1.000
Mechanical ventilation, days	3.0 (1.0–5.0)	2.0 (1.0–3.0)	0.042	3.0 (2.0–5.0)	2.0 (1.0–3.0)	0.048
Cardiopulmonary resuscitation, *n* (%)	19 (21.3)	6 (17.1)	0.782	6 (16.7)	6 (17.1)	1.000
Evidence of aspiration, *n* (%)	8 (9.0)	5 (13.9)	0.595	4 (11.1)	5 (13.9)	1.000
Oto score	3.0 (1.0–4.0)	3.0 (1.0–4.0)	0.856	2.0 (1.0–3.5)	3.0 (1.0–4.0)	0.238
Machine perfusion, *n* (%)	3 (3.4)	0 (0.0)	0.526	1 (2.8)	0 (0.0)	1.000

Values are expressed as median (interquartile range) or number (percentage), as appropriate.

PD had greater height and weight compared with NPD, however, BMI did not differ significantly between the two groups, with median values of 24.2 kg/m^2^ (IQR 22.8–26.0) in Group PD and 23.5 kg/m^2^ (IQR 21.8–26.1) in group NPD (*p* = 0.447). Smoking history was comparable between groups (*p* = 0.958). Donor oxygenation at the time of evaluation was also similar, with a median PaO_2_/FiO_2_ ratio of 476.2 (IQR 433.7–550.5) in PD and 487.0 (IQR 441.0–535.0) in NPD (*p* = 0.978). Pulmonary contusions on chest imaging were present in 34 PD (94.4%) compared with 1 NPD (1.1%, *p* < 0.001). The prevalence of bronchoscopic secretions was similar between groups, occurring in 4 PD (11.4%) and 11 NPD (12.5%; *p* > 0.99). The duration of mechanical ventilation prior to procurement was shorter in PD, with a median of 2.0 days (IQR 1.0–3.0) compared with 3.0 days (IQR 1.0–5.0) in NPD (*p* = 0.042). Cardiopulmonary resuscitation was performed in 6 PD (17.1%) and 19 NPD (21.3%; *p* = 0.782), while evidence of aspiration was observed in 5 PD (13.9%) and 8 NPD (9.0%; *p* = 0.595). Finally, donor quality as assessed by the Oto score was comparable between groups, with a median Oto score of 3.0 (IQR 1.0–4.0) in both PD and NPD (*p* = 0.856). The number of donor lungs evaluated using EVLP did not differ between groups.

### 3.2 Recipient analysis

Recipients' characteristics are shown in Table 2. Female recipients were 35 (39.3%) in NPD and 11 PD recipients (30.6%, *p* = 0.474). PD recipients were younger, with a median age of 51.5 years (IQR 42.8–59.0), compared with NPD (57.0 years, IQR 49.0–62.0, *p* = 0.027). Recipient anthropometric characteristics were comparable between groups. BMI did not differ significantly, with median values of 25.0 kg/m^2^ (IQR 20.5–28.1) in PD recipients and 23.9 kg/m^2^ (IQR 20.9–27.2) in NPD recipienst (*p* = 0.586). The distribution of underlying lung diseases did not differ significantly between groups (*p* = 0.335). Interstitial lung disease was the most common indication for transplantation in both groups, affecting 37 NPD recipients (41.6%) and 15 PD recipients (41.7%). Pre-transplant priority status, as assessed by the LAS, was comparable between groups, with median values of 32.0 (IQR 20.0–35.1) in NPD recipients and 30.7 (IQR 9.0–32.5) in PD recipients (*p* = 0.258). The prevalence of pre-existing comorbidities was similar between groups. Pulmonary hypertension was present in 15 NPD (16.9%) and 6 PD (16.7%; *p* > 0.99) recipients.

**Table 2:** Recipient characteristics and perioperative variables according to donor trauma status.

	Overall cases (*n* = 125)			Propensity score-matched pairs (*n* = 72)		
Variables	No Polytrauma	Polytrauma	*p*	No Polytrauma	Polytrauma	*p*
	*N* = 89	*N* = 36		*N* = 36	*N* = 36	
Age, years	57.0 (49.0–62.0)	51.5 (42.8–59.0)	0.027	55.0 (40.8–61.2)	51.5 (42.8–59.0)	0.681
Female sex, *n* (%)	35 (39.3)	11 (30.6)	0.474	17 (47.2)	11 (30.6)	0.227
Blood type, *n* (%)			0.253			0.620
A	48(53.9)	16 (44.4)		20 (55.6)	16 (44.4)	
B	7 (7.9)	8 (22.2)		4 (11.1)	8 (22.2)	
O	29 (32.6)	10 (27.8)		10 (27.8)	10 (27.8)	
AB	5 (5.6)	2 (5.6)		2 (5.6)	2 (5.6)	
Height, cm	68.0 (56.0–80.0)	72.5 (59.8–84.2)	0.208	60.5 (52.8–73.0)	72.5 (59.8–84.2)	0.026
Weight, kg	167.0 (161.0–175.0)	171.5 (166.5–178.0)	0.053	167.0 (160.8–172.2)	171.5 (166.5–178.0)	0.065
Body Mass Index, kg/m^2^	23.9 (20.9–27.2)	25.0 (20.5–28.1)	0.586	22.1 (19.1–24.3)	25.0 (20.5–28.1)	0.049
Underlying diagnosis, *n* (%)			0.335			0.526
Interstitial lung disease	37 (41.6)	15 (41.7)		11 (30.6)	15 (41.7)	
Chronic obstructive pulmonary disease	23 (25.8)	7 (19.4)		10 (27.8)	7 (19.4)	
Cystic fibrosis	13 (14.6)	6 (16.7)		8 (22.2)	6 (16.7)	
Chronic lung allograft dysfunction	1 (1.1)	2 (5.6)		1 (2.8)	2 (5.6)	
Hypersensitivity pneumonitis	3 (3.4)	0 (0.0)		0 (0.0)	0 (0.0)	
Sarcoidosis	0 (0.0)	2 (5.6)		0 (0.0)	2 (5.6)	
Other^*^	12 (13.5)	4 (11.1)		6 (16.7)	4 (11.1)	
Lung allocation score	32.0 (20.0–35.1)	30.7 (9.0–32.5)	0.258	32.0 (15.5–35.1)	30.7 (9.0–32.5)	0.549
Systemic arterial hypertension, *n* (%)	27 (30.3)	6 (16.7)	0.178	9 (25.0)	6 (16.7)	0.562
Cardiovascular disease, *n* (%)	11 (12.4)	4 (11.1)	1.000	3 (8.3)	4 (11.1)	1.000
Renal disease, *n* (%)	10 (11.2)	4 (11.1)	1.000	2 (5.6)	4 (11.1)	0.670
Gastrointestinal disease, *n* (%)	18 (20.2)	3 (8.3)	0.178	8 (22.2)	3 (8.3)	0.190
Osteoporosis	28 (31.5)	14 (38.9)	0.557	12 (33.3)	14 (38.9)	0.806
Psychiatric disease, *n* (%)	5 (5.6)	0 (0.0)	0.343	4 (11.1)	0 (0.0)	0.123
Diabetes, *n* (%)	14 (15.7)	6 (16.7)	1.000	7 (19.4)	6 (16.7)	1.000
Obesity, *n* (%)	5 (5.6)	3 (8.3)	0.874	3 (8.3)	3 (8.3)	1.000
Chronic infection, *n* (%)	13 (14.6)	9 (25.0)	0.262	8 (22.2)	9 (25.0)	1.000
Pulmonary hypertension, *n* (%)	15 (16.9)	6 (16.7)	1.000	6 (16.7)	6 (16.7)	1.000
Type of transplant, *n* (%)			0.514			0.414
Single	24 (27.0)	7 (19.4)		11 (30.6)	7 (19.4)	
Bilateral	65 (73.0)	29 (80.6)		25 (69.4)	29 (80.6)	
Urgent transplantation, *n* (%)	13 (14.6)	6 (16.7)	0.988	7 (19.4)	6 (16.7)	1.000
Graft size reduction, *n* (%)	11 (12.4)	4 (11.1)	1.000	3 (8.3)	4 (11.1)	1.000
Preoperative ECLS, *n* (%)	9 (10.1)	2 (5.6)	0.641	4 (11.1)	2 (5.6)	0.670
Intraoperative ECLS, *n* (%)	23 (25.8)	12 (33.3)	0.532	7 (19.4)	12 (33.3)	0.285
1st lung ischemia time, min	270.0 (225.0–360.0)	265.0 (225.0–309.0)	0.275	267.5 (214.8–346.2)	265.0 (225.0–309.0)	0.888
2nd lung ischemia time, min	430.0 (375.0–510.0)	420.0 (345.0–480.0)	0.285	395.0 (340.0–450.0)	420.0 (345.0–480.0)	0.828

Values are expressed as median (interquartile range) or number (percentage), as appropriate.

^*^Other diagnoses include combined pulmonary fibrosis-emphysema, pulmonary hemosiderosis, bronchiectasis, SARS-CoV-2-related lung disease, lymphangioleiomyomatosis, primary ciliary dyskinesia, graft vs. host disease. ECLS: extracorporeal life support.

Single lung transplantation was performed in 24 NPD (27.0%) and 7 PD (19.4%) recipients, whereas bilateral lung transplantation was performed in 65 (73.0%) and 29 (80.6%) recipients, respectively (*p* = 0.514). Urgent transplantation was required in 13 NPD (14.6%) and 6 PD recipients (16.7%; *p* = 0.988). Graft size reduction was performed in 11NPD recipients (12.4%) and in 4 PD recipients (11.1%, *p* = 1.000). Extracorporeal life support (ECLS) prior to transplantation was used in 9 NPD (10.1%) and 2 PD (5.6%; *p* = 0.641) recipients, while intraoperative ECLS was required in 23 (25.8%) and 12 (33.3%) recipients, respectively (*p* = 0.532). Cold ischemia time for the first lung was similar between groups, with median values of 270 min (IQR 225.0–360.0) in NPD and 265.0 min (IQR 225.0–309.0) in PD (*p* = 0.275) recipients. Likewise, cold ischemia time for the second lung did not differ significantly, with median values of 430.0 min (IQR 375.0–510.0) and 420.0 min (IQR 345.0–480.0), respectively (*p* = 0.285).

### 3.3 Early postoperative outcomes

Early postoperative outcomes were comparable between recipients transplanted with lungs from PD and NPD (Table 3). The duration of postoperative mechanical ventilation did not differ significantly between groups, with a median of 101.0 h (IQR 36.5–204.5) in PD and 126.5 h (IQR 32.8–358.0) in NPD (*p* = 0.730) recipients. Similarly, ICU length of stay was comparable, with a median of 6.9 days (IQR 4.0–10.7) in PD and 8.9 days (IQR 5.9–17.1) in NPD (*p* = 0.167) recipients. Postoperative use of noninvasive ventilation (NIV) occurred in 2 PD (8.7%) and 8 NPD (14.3%, *p* = 0.759) recipients. Lactate levels measured 3 h after ICU admission were similar between groups, with median values of 4.5 mmol/L (IQR 3.2–6.1) in PD and 3.9 mmol/L (IQR 2.7–5.8) in NPD (*p* = 0.197) recipients. Reoperation for postoperative bleeding occurred only in NPD group (3 recipients, 5.5%, *p* = 0.619). Hemodynamic support was comparable between groups, with median values of 16.0 (IQR 8.5–32.5) in PD group and 26.5 (IQR 9.5–46.2) in NPD group (*p* = 0.157). The incidence of AKI was similar between groups, occurring in 6 PD recipients (26.1%) and 14 NPD recipients (25.0%; *p* > 0.99). Peak serum creatinine levels were also comparable (*p* = 0.126). Tracheostomy was required in 5 PD recipients (21.7%) and 17 NPD recipients (30.4%, *p* = 0.617), while CRRT was used in 1 (6.2%) and 5 (12.5%) recipients, respectively (*p* = 0.838). Postoperative delirium occurred in 6 PD recipients (26.1%) and 17 NPD recipients (30.4%; *p* = 0.915). The incidence and severity of PGD at 72 h were similar between groups (*p* = 0.839). Surgical complications requiring reoperation occurred in 11 PD (31.4%) and 17 NPD (19.8%; *p* = 0.254) recipients. Bronchial complications were observed in 10 PD recipients (27.8%) and 12 NPD recipients (13.5%), with bronchial stenosis being the most frequent event; however, overall distribution did not differ significantly between groups (*p* = 0.294). Total hospital LOS was similar between groups, with a median of 38.0 days (IQR 29.8–61.2) in PD group and 35.0 days (IQR 27.0–52.0) in NPD group (*p* = 0.183). These findings were confirmed in the PSM cohort, where no significant differences were observed between groups across all evaluated early postoperative variables.

**Table 3:** Early postoperative outcomes according to donor trauma status.

	Overall cases (*n* = 125)			Propensity score-matched pairs (*n* = 72)		
Variables	No Polytrauma	Polytrauma	*p*	No Polytrauma	Polytrauma	*p*
	*N* = 89	*N* = 36		*N* = 36	*N* = 36	
Mechanical ventilation, hours	126.5 (32.8–358.0)	101.0 (36.5–204.5)	0.730	72.0 (34.0–233.0)	101.0 (36.5–204.5)	0.792
ICU length of stay, days	8.9 (5.9–17.1)	6.9 (4.0–10.7)	0.167	7.7 (4.7–13.2)	6.9 (4.0–10.7)	0.590
Post-operative NIV, *n* (%)	8 (14.3)	2 (8.7)	0.759	4 (17.4)	2 (8.7)	0.662
Lactate at ICU admission (3 h), mmol/L	3.9 (2.7–5.8)	4.5 (3.2–6.1)	0.197	3.9 (2.8–5.3)	4.5 (3.2–6.1)	0.249
Inotropic score	26.5 (9.5–46.2)	16.0 (8.5–32.5)	0.157	25.0 (3.0–41.5)	16.0 (8.5–32.5)	0.757
AKI, *n* (%)	14 (25.0)	6 (26.1)	>0.99	3 (13.0)	6 (26.1)	0.457
Peak serum creatinine, mg/dL	1.0 (0.7–1.5)	1.4 (0.9–2.0)	0.126	0.8 (0.6–1.2)	1.4 (0.9–2.0)	0.007
Tracheostomy, *n* (%)	17 (30.4)	5 (21.7)	0.617	5 (21.7)	5 (21.7)	1,000
CRRT, *n* (%)	5 (12.5)	1 (6.2)	0.838	1 (5.6)	1 (6.2)	1,000
Delirium, *n* (%)	17 (30.4)	6 (26.1)	0.915	6 (26.1)	6 (26.1)	1,000
PGD at 72 h, *n* (%)			0.839			0.667
Grade 3	29 (32.6)	10 (27.8)		8 (22.2)	10 (27.8)	
Grade 2	22 (24.7)	10 (27.8)		10 (27.8)	10 (27.8)	
Grade 1	16 (18.0)	5 (13.9)		9 (25.0)	5 (13.9)	
Grade 0	22 (24.7)	11 (30.6)		9 (25.0)	11 (30.6)	
Reoperation for bleeding, *n* (%)	3 (5.5)	0 (0.0)	0.619	1 (4.5)	0 (0.0)	0.982
Other complications requiring reoperation, *n* (%)	17 (19.8)	11 (31.4)	0.254	9 (26.5)	11 (31.4)	0.851
Bronchial complications, *n* (%)			0.294			0.697
Stenosis	8 (9.0)	7 (19.4)		4 (11.1)	7 (19.4)	
Unilateral dehiscence	3 (3.4)	2 (5.6)		1 (2.8)	2 (5.6)	
Bilateral dehiscence	1 (1.1)	1 (2.8)		1 (2.8)	1 (2.8)	
None	77 (86.5)	26 (72.2)		30 (83.3)	26 (72.2)	
Total hospital stay, days	35.0 (27.0–52.0)	38.0 (29.8–61.2)	0.183	34.5 (27.0–50.2)	38.0 (29.8–61.2)	0.211

Values are expressed as median (interquartile range) or number (percentage), as appropriate. ICU, intensive care unit; NIV, noninvasive ventilation; AKI, acute kidney injury; CRRT, continuous renal replacement therapy; PGD, primary graft dysfunction.

### 3.4 Mid-term outcomes

**Figure 1:**
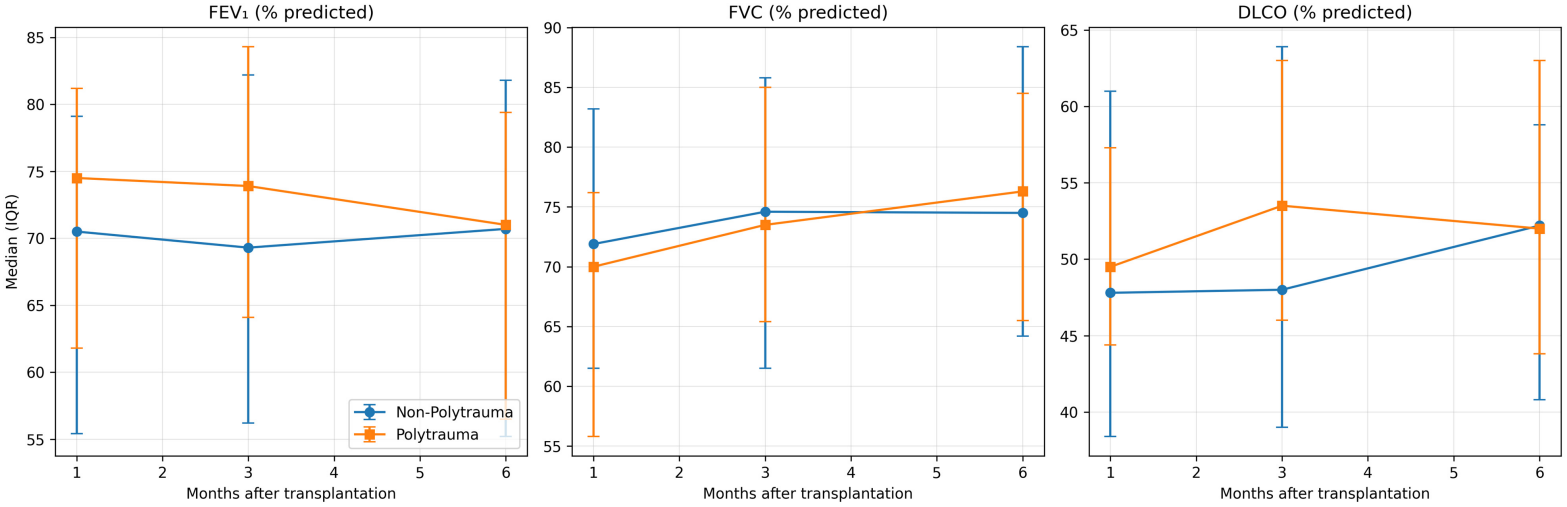
Post-transplant pulmonary function over time according to donor trauma status. Median (IQR) values of FEV1, FVC, and DLCO, expressed as percentage of predicted, at 1, 3, and 6 months after lung transplantation in recipients of lungs from polytrauma and non-polytrauma donors.

Post-transplant pulmonary function did not differ significantly between PD and NPD recipients at any of the evaluated time points (Table 4, Figure 1). At 1 month after transplantation, median FEV1 was 74.5% of predicted in PD group and 70.5% in the NPD group (*p* = 0.488). Median FVC values were 70.0 and 71.9%, respectively (*p* = 0.351), while median VC was 67.5% in PD group and 71.9% in NPD group (*p* = 0.242). DLCO was comparable between groups, with median values of 49.5 and 47.8%, respectively (*p* = 0.612). TLC was also similar, with median values of 80.7% in PD group and 83.3% in NPD group (*p* = 0.119). At 3 and 6 months, pulmonary function parameters remained comparable. The incidence of CLAD was similar between groups: 14 recipients (38.9%) in PD recipients and 28 NPD recipients (31.5%, *p* = 0.557).

**Table 4:** Post-transplant pulmonary function and mid-term graft outcomes.

	Overall cases (*n* = 125)			Propensity score-matched pairs (*n* = 72)		
Variables	No Polytrauma	Polytrauma	*p*	No Polytrauma	Polytrauma	*P*
	*N* = 89	*N* = 36		*N* = 36	*N* = 36	
Pulmonary function at 1 month						
FEV1%	70.5 (55.4–79.1)	74.5 (61.8–81.2)	0.488	69.5 (53.3–78.0)	74.5 (61.8–81.2)	0.245
FVC%	71.9 (61.5–83.2)	70.0 (55.8–76.2)	0.351	70.0 (56.8–79.0)	70.0 (55.8–76.2)	0.721
VC%	71.9 (58.5–81.0)	67.5 (53.8–74.4)	0.242	72.1 (59.1–77.3)	67.5 (53.8–74.4)	0.354
DLCO%	47.8 (38.4–61.0)	49.5 (44.4–57.3)	0.612	53.0 (39.0–63.0)	49.5 (44.4–57.3)	0.765
TLC%	83.3 (72.9–101.2)	80.7 (70.8–86.0)	0.119	85.0 (69.9–100.8)	80.7 (70.8–86.0)	0.143
Pulmonary function at 3 months						
FEV1%	69.3 (56.2–82.2)	73.9 (64.1–84.3)	0.250	69.3 (60.5–82.8)	73.9 (64.1–84.3)	0.561
FVC%	74.6 (61.5–85.8)	73.5 (65.4–85.0)	0.992	77.8 (67.8–88.4)	73.5 (65.4–85.0)	0.310
VC%	72.0 (60.0–82.0)	70.0 (63.0–81.3)	0.866	78.0 (69.5–84.9)	70.0 (63.0–81.3)	0.178
DLCO%	48.0 (39.0–63.9)	53.5 (46.0–63.0)	0.461	55.0 (45.5–66.5)	53.5 (46.0–63.0)	0.471
TLC%	84.2 (70.5–102.8)	77.9 (69.7–87.9)	0.300	90.5 (79.1–103.5)	77.9 (69.7–87.9)	0.033
Pulmonary function at 6 months						
FEV1%	70.7 (55.2–81.8)	71.0 (56.5–79.4)	0.914	70.7 (57.0–80.9)	71.0 (56.5–79.4)	0.954
FVC%	74.5 (64.2–88.4)	76.3 (65.5–84.5)	0.770	74.5 (70.2–87.6)	76.3 (65.5–84.5)	0.960
VC%	75.0 (63.9–87.8)	72.5 (61.0–86.0)	0.724	76.0 (68.4–87.8)	72.5 (61.0–86.0)	0.430
DLCO%	52.2 (40.8–58.8)	52.0 (43.8–63.0)	0.589	54.5 (39.3–61.2)	52.0 (43.8–63.0)	0.886
TLC%	80.0 (64.0–98.9)	77.2 (70.7–97.8)	0.996	83.5 (72.0–98.2)	77.2 (70.7–97.8)	0.450
Mid-term graft outcomes						
CLAD	28 (31.5)	14 (38.9)	0.557	16 (44.4)	14 (38.9)	0.811
30-days mortality, %	16 (18)	2 (5.6)	0.083	2 (5.6)	2 (5.6)	0.999
6-months mortality, %	24 (27)	3 (8.3)	0.048	2 (5.6)	3 8.3	0.643
Retransplantation, *n* (%)	6 (6.7)	1 (2.8)	0.672	5 (13.9)	1 (2.8)	0.205

Values are expressed as median (interquartile range) or number (percentage), as appropriate. Pulmonary function parameters are expressed as percentage of predicted values. DLCO, diffusing capacity of the lung for carbon monoxide; FEV1, forced expiratory volume in one second; FVC, forced vital capacity; TLC, total lung capacity; CLAD, chronic lung allograft dysfunction.

**Figure 2:**
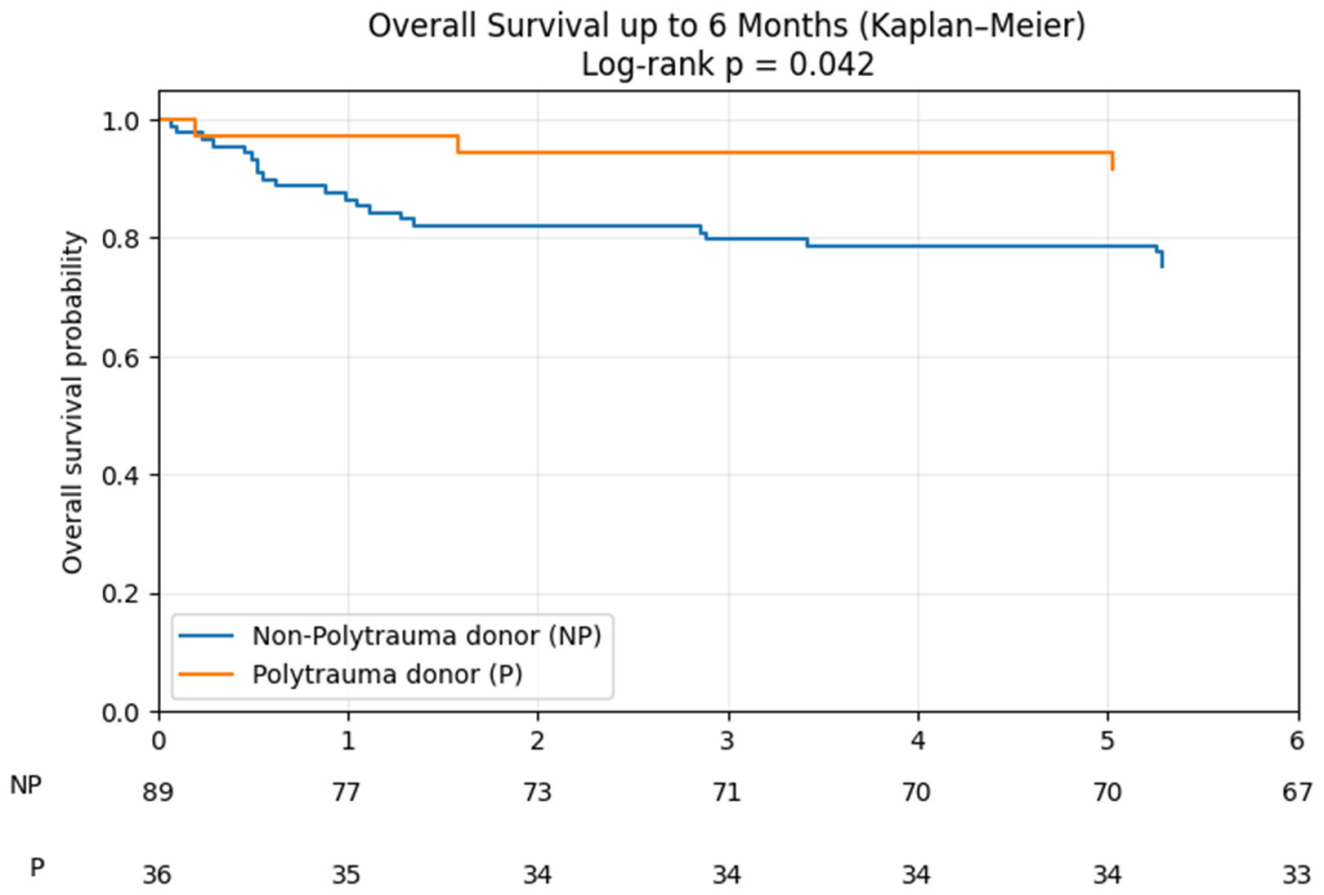
Kaplan Meier 6-month overall survival analysis according to donor trauma status. Numbers at risk are shown below the *x*-axis.

At 30 days after lung transplantation, OS was high in both groups, with 2 deaths (5.6%) in the PD group and 16 deaths (18.0%) in the unmatched NPD group. In the unmatched cohort, 6-month OS was 91.7% in PD recipients (3 deaths) compared with 75.3% in NPD (24 deaths; log-rank *p* = 0.042) (Figure 2). However, this difference was not confirmed after PSM, where no significant differences in 6-month survival were observed between the two groups [PD: 3 deaths (8.3%) vs. Matched NPD: 2 deaths (5.6%); log-rank *p* = 0.66].

To further validate these findings, a multivariable Cox proportional hazards model was performed on the entire cohort, adjusting for donor age, recipient age, and ischemic time. The analysis confirmed that polytrauma was not an independent predictor of mortality (HR 0.77; 95% CI 0.44–1.37; *p* = 0.382). In this model, only total ischemic time showed a significant association with survival (HR 1.00; 95% CI 1.00–1.00; *p* = 0.048).

Similarly, in the matched cohort, perioperative outcomes, including PGD rates, ICU stay, and complication rates, remained comparable between groups. Pulmonary function at 1, 3, and 6 months, as well as CLAD incidence, did not differ significantly between PD and NPD recipients.

## 4 Discussion

In this study, we evaluated the impact of donor polytrauma on early and mid-term outcomes after lung transplantation. In the unmatched analysis, recipients of lungs from PD showed a higher 6-month survival compared with NPD. However, this difference was no longer observed after PSM, suggesting that the apparent survival advantage was primarily driven by baseline differences, particularly donor and recipient age.

Importantly, after adjustment for these confounders, outcomes remained comparable between groups across all evaluated domains, including perioperative course, PGD incidence, and pulmonary function recovery.

To further scrutinize the impact of donor polytrauma, we employed a multivariable Cox proportional hazards model. This analysis confirmed that when adjusting for donor and recipient age and ischemic time, polytrauma was not an independent predictor of mortality (HR 0.77; 95% CI 0.44–1.37; *p* = 0.382). These findings support the concept that trauma-related lung injury, when appropriately selected, does not adversely affect transplant outcomes and that the survival advantage initially observed in the unmatched cohort (*p* = 0.042) was likely driven by the more favorable baseline profile of polytrauma donors, such as younger age. In fact, PD in our cohort were significantly younger and had fewer comorbidities, with shorter ICU stays prior to procurement, features that are consistently reported in trauma-related donation and are known to be associated with better graft quality. Similar demographic profiles of trauma donors have been described in other studies, where younger age and limited pre-existing diseases were common features despite the presence of acute lung injury or contusions at imaging (2, 16).

Younger donor lungs are known to exhibit greater structural and functional resilience, including better alveolar–capillary integrity, reduced endothelial dysfunction, and preserved regenerative capacity, all of which may mitigate susceptibility to ischemia–reperfusion injury after transplantation. In addition, a shorter ICU-stay before organ procurement likely reflects reduced exposure to potentially injurious factors such as prolonged mechanical ventilation, ventilator-associated lung injury and infection (17). Finally, the lower burden of comorbidities typically observed in PD, such as smoking-related lung disease, cardiovascular disease, or metabolic disorders, may further contribute to improved graft quality, due to the lower pre-existing endothelial and microvascular dysfunction. Collectively, these donor-related characteristics may confer protection against the development of PGD. These concepts are supported by donor risk stratification models, including the Oto score, in which younger age, preserved oxygenation, and limited ICU-stay are weighted as favorable prognostic factors for lung graft utilization and outcomes (9).

Historically, lungs from polytrauma donors have been considered marginal primarily because of the high incidence of pulmonary contusions, aspiration, and inflammation, factors traditionally associated with PGD. Consequently, most available studies have focused almost exclusively on PGD as the main outcome of interest. Diamond et al. (18) established PGD as a key early endpoint after LTX and highlighted donor-related risk factors, including trauma and hypoxemia. However, subsequent analyses have yielded conflicting results regarding the independent role of trauma, suggesting that trauma may act more as a surrogate for PGD rather than a determinant of irreversible graft damage. In our cohort, key donor parameters at the time of organ offer, including PaO_2_/FiO_2_ ratio, Oto score, and duration of mechanical ventilation, were optimal in PDs and largely overlapping with those observed in NPDs. Despite a markedly higher prevalence of pulmonary contusions in PDs, no significant differences were observed in perioperative outcomes, such as rates or severity of PGD at 72 h, need for postoperative ECMO, ICU length of stay, or early complications. This observation supports the concept that lung contusions alone should not be regarded as an absolute marker of donor marginality. While trauma-related lung injury has traditionally raised concerns about an increased risk of PGD, which was not observed in our study, our data underscore the importance of refined donor selection rather than blanket exclusion. Specifically, the use of lungs from trauma donors with limited duration of intubation, preserved oxygenation, younger age, and favorable global donor risk scores appear to mitigate potential risks associated with pulmonary contusions, as already seen by Alisha et al. (19). After adjustment for baseline differences, lung grafts from PD demonstrate outcomes comparable to those from NPD. These findings support the safe and selective use of such grafts as part of donor pool expansion strategies.

Importantly, functional outcomes assessed by serial spirometry up to 6 months were comparable between groups, with no differences at any evaluated time point. These non-significant findings provide strong evidence of clinical equivalence between lungs procured from PDs and NPDs. This aspect represents a major strength of our study and addresses a key gap in literature. While previous reports have largely limited outcome assessment to PGD and short-term outcomes, data on postoperative lung function are scarce. Our results indicate that early radiological abnormalities related to trauma do not translate into impaired functional recovery, supporting the concept that pulmonary contusions are often reversible when appropriate donor management and recipient selection are applied (20, 21). This is consistent with experimental and clinical evidence showing rapid resolution of trauma-related lung injury under protective ventilation strategies (22).

The study's retrospective and single-center design may limit the generalizability of the findings and precludes definitive causal inference. As highlighted by the lack of significant difference in our adjusted models, PD in our series often represent a “best-case” subgroup, characterized by younger age and fewer comorbidities, which may compensate for the initial traumatic insult.

Regarding the longitudinal data, the exclusion of a small subset of cases (*n* = 16) due to missing donor or functional parameters was addressed through sensitivity analyses. These confirmed that the missingness occurred at random and did not systematically favor better-quality donors, as baseline characteristics (age, LAS, and diagnosis) were comparable between included and excluded patients, thereby maintaining the representativeness of the analyzed cohort. Furthermore, the absence of standardized trauma severity scores and quantitative grading of pulmonary contusions may have introduced heterogeneity within the polytrauma donor group and potentially biased the results toward more favorable outcomes, as donors with less severe thoracic injuries and preserved functional parameters were more likely to be accepted for transplantation. Although formal injury severity scales were unavailable, indirect indicators such as shorter duration of mechanical ventilation and preserved oxygenation suggest that the included polytrauma donors represented a relatively selected subgroup.

In conclusion, our study highlights that lung grafts from PD are an underutilized resource with favorable biological characteristics. After adjusting for potential selection bias through PSM, we observed excellent short-term survival and functional outcomes, with no significant differences compared to NPD. These findings support the continued use of traumatized grafts and a more nuanced evaluation of PD; their exclusion based solely on imaging findings may be unwarranted. Ultimately, following a rigorous selection and management protocol allows for the safe expansion of the donor pool without compromising transplant success.

## Data Availability

The raw data supporting the conclusions of this article will be made available by the authors, without undue reservation.
